# Echocardiographic Prediction of Left Ventricular Dysfunction After Transcatheter Patent Ductus Arteriosus Closure in Children

**DOI:** 10.3389/fped.2019.00409

**Published:** 2019-10-15

**Authors:** Miao Hou, Weiguo Qian, Bo Wang, Wanping Zhou, Jianmin Zhang, Yueyue Ding, Qiuqin Xu, Jie Huang, Jie Shen, Lei Cao, Haitao Lv, Ling Sun

**Affiliations:** Department of Cardiology, Children's Hospital of Soochow University, Suzhou, China

**Keywords:** patent ductus arteriosus, transcatheter closure, left ventricular dysfunction, echocardiography, children

## Abstract

**Objectives:** To evaluate the change of left ventricular (LV) systolic function after transcatheter patent ductus arteriosus (PDA) closure in children, and to identify whether echocardiography parameters could be the predictors of LV dysfunction post-PDA closure if present.

**Methods:** This study enrolled 191 pediatric PDA patients, and all of them underwent successful transcatheter PDA closure between January 2016 and December 2018. The patent ductus arteriosus diameter (PDAd), aortic root diameter (AOd), left atrial diameter (LAd), right ventricular outflow tract dimension (RVOT), LV end-diastolic dimension (LVEDD), and LV end-systolic dimension (LVESD) were all measured by echocardiography at pre-closure, post-closure (within 24 h after the procedure), and follow-up (3 months after the procedure). The ratio of PDAd to AOd (PDAd/AOd), the ratio of LAd to AOd (LAd/AOd), the left ventricular ejection fraction (LVEF), and the fractional shortening (FS) were calculated.

**Results:** The LAd, LVESD, LVEDD, FS, and LVEF decreased significantly in the 24 h after closure, compared to pre-closure levels. However, all echocardiography parameters recovered to pre-closure levels at 3 months after PDA closure in all patients. Moreover, the pre-closure LAd, LVEF, PDAd/AOd, and LAd/AOd were higher in the patients with post-closure LV systolic dysfunction than in those without post-closure LV systolic dysfunction. Furthermore, the pre-closure LVEF, PDAd/AOd, and LAd/AOd were correlated with the post-closure LVEF, and pre-closure LVEF ≤ 66.5%, PDAd/AOd ≥ 0.28, and LAd/AOd ≥ 1.54 predict the post-closure LV systolic dysfunction.

**Conclusion:** Transcatheter closure of PDA causes a significant deterioration in LV systolic function early after PDA closure, which recovered completely within 3 months of post-closure in children. Pre-closure LVEF, PDAd/AOd, and LAd/AOd can be the predictors of post-closure left ventricular systolic dysfunction.

## Introduction

Patent ductus arteriosus (PDA) is a common form of congenital heart disease with a left-to-right shunt (1). It has a broad spectrum of clinical manifestations, and the natural history of PDA mainly depends upon its size. Hemodynamically significant PDA leads to a left ventricle (LV) volume overload and remodeling and ultimately leads to severe complications, such as congestive heart failure, Eisenmenger's syndrome, atrial arrhythmias, endarteritis, and ductus aneurysm (2–4).

Transcatheter closure of PDA has been in development for nearly 30 years. Now, transcatheter PDA closure has been proven to be safe and effective with short- and long-term results comparable to surgical closure, and it has become the leading approach to the closure of most instances of PDA (5). Recently, several reports demonstrated that left ventricular systolic properties altered in adult PDA patients (6, 7), and PDA closure also led to an immediate deterioration of LV systolic function in children (8, 9). However, pre-closure predictors of the LV systolic dysfunction after PDA closure have not yet been clearly demonstrated.

Echocardiography is the most common diagnostic method that provides information regarding PDA size and hemodynamics, and it is also the most frequently used method for the evaluation of cardiac chamber size and LV systolic performance (10). Moreover, these parameters obtained by echocardiography correlate well with those measured during cardiac catheterization and radionuclide angiography (11, 12), and the possibility of the use of echocardiographic parameters in risk assessment for adverse cardiac events has been proven in recent studies (13–15). Therefore, the present study aimed to investigate the changes of LV systolic function after PDA closure, and to identify whether echocardiography indicators, if present, could be the predictors of LV dysfunction after PDA closure.

## Methods

### Study Population

The study was approved by the institutional ethics committee at the Children's Hospital of Soochow University, and written informed consent was obtained from the patients' parents in all cases.

This study enrolled 191 children. All of them were diagnosed with isolated PDA and underwent successful transcatheter PDA closure between January 2016 and December 2018 in the cardiology department of the Children's Hospital of Soochow University. Patients of unsuitable PDA size for interventional closure were excluded.

### Echocardiography

All echocardiographic examinations were carried out using the General Electric (GE) VIVID 7 ultrasound (Horten, Norway) with M4S and 5S probe and the GE EchoPAC workstation (BT 09, Horten, Norway). Some PDA children required sedation for echocardiography examination. For each PDA patient, echocardiography examinations were conducted at pre-closure, post-closure (within 24 h after PDA closure), and follow-up (3 months after PDA closure), respectively.

The patent ductus arteriosus diameter (PDAd) was measured in the high left parasternal short-axis view. The aortic root diameter (AOd), left atrial diameter (LAd), right ventricular outflow tract dimension (RVOT), LV end-diastolic dimension (LVEDD), and LV end-systolic dimension (LVESD) were obtained from the parasternal long-axis view. From these measurements, the following LV parameters were calculated: Fractional shortening $\left(FS\right)\;=\frac{\text{LVEDD}-\text{LVESD}}{\text{LVESD}}\times 100$, and LV ejection fraction (EF) = $\left(\text{EF}\right)=\frac{\text{LVEDD volume}-\text{LVESD volume}}{\text{LVEDD volume}}\;\times \;100$, where $\text{LVEDD volume }=\;\frac{7\;\times \;LVEDD^{3}}{2.4\;+\text{ LVEDD}}$ and $\text{LVESD volume }=\;\frac{7\;\times \;LVESD^{3}}{2.4\;+\text{ LVESD}}$ (16). To normalized echocardiographic indicators, the ratio of LAd to AOd (LAd/AOd) as well as the ratio of PDAd to AOd (PDAd/AOd) was calculated. LV systolic dysfunction was defined as a post-PDA closure LVEF of <55% (8).

### Cardiac Catheterization

Midazolam, ketamine, or propofol were used for sedation in all patients, and a dose of 100 unit/kg of heparin was given after vein and artery puncture. The lateral aortogram was performed at the distal aortic arch before PDA closure. Transcatheter closure of PDA was performed using an antegrade or retrograde technique. An additional aortogram was performed to confirm complete shutdown after the procedure.

### Statistical Analysis

Data are expressed as mean ± standard deviation. Changes in echocardiographic parameters were analyzed with paired *t*-test. The correlation between two continuous variables was determined using linear regression analysis. Multiple stepwise linear regression analyses were used to identify pre-closure echocardiography indicators of post-closure LV systolic dysfunction. Firstly, several statistically significant risk factors were screened out with univariate analysis, and *P* < 0.05 was considered statistically significant. Afterwards, multivariate analysis was performed using variables that were significant on univariate analysis, and *P* < 0.1 was considered significant. Receiver operating characteristic (ROC) analysis was used to find optimal cut-offs for each parameter for when the post-closure LVEF was below 55%. All of the statistical analyses used the Statistical Package for the Social Sciences (SPSS), version 19.0 for Windows (SPSS, Chicago, IL, USA).

## Result

### Clinical Characteristics of Patients

The clinical features of these PDA patients are described in Table 1. There were 60 boys and 131 girls in this study, and the median age was 23 months (range 3–184 months) at the time of the procedure. The PDA pulmonic end size was 3.06 ± 1.25 mm. The closure was achieved in all 191 patients who underwent transcatheter, and Amplazter duct occluders were used in all cases. The heart rate, systolic blood pressure, and diastolic blood pressure levels of PDA patients were similar between pre- and post-closure (Supplemental Table 1).

**Table 1:** Clinical data of 191 children undergoing PDA occlusion.

	**PDA patients (*n* = 191)**
Age (month)	33.71 ± 32.68
Gender (Boys/Girls)	60/131
Weight (kg)	8.10 ± 2.34
PDA diameter (mm)	3.06 ± 1.25

### The Comparison of Echocardiography Parameters Between Pre-closure and Post-closure

The LAd, LVESD, LVEDD, FS, and LVEF significantly decreased within 24 h after closure compared to pre-closure levels, while there was no difference in AOd and RVOT values between pre- and post-closure.

Of the 191 children, 27 of them showed LV systolic dysfunction within 24 h of PDA closure. The pre-closure LVEF, LAd, PDAd/AOd, and LAd/AOd were higher in the patients with post-closure LV systolic dysfunction than those without post-closure LV systolic dysfunction (Table 2).

**Table 2:** Comparison of echocardiography parameters at pre-closure, post-PDA closure and follow up (*n* = 191).

	**Pre-closure**	**Post-closure 24 h**	**Follow up post-closure 3 months**
AOd (mm)	14.24 ± 3.45	14.26 ± 2.77	14.14 ± 3.57
Lad (mm)	19.86 ± 3.95	18.63 ± 3.57^*^	19.54 ± 5.39
LVESD (mm)	21.19 ± 7.68	20.28 ± 5.37^*^	21.81 ± 8.62
LVEDD (mm)	35.61 ± 6.46	34.48 ± 7.17^*^	35.37 ± 8.31
RVOT (mm)	18.46 ± 3.63	18.03 ± 4.14	18.37 ± 4.11
FS (%)	38.81 ± 4.32	34.55 ± 4.76^*^	38.38 ± 4.78
LVEF (%)	70.03 ± 5.07	65.55 ± 6.61^*^	70.29 ± 5.36

* *P <0.05 vs. pre-closure*.

*AOd, Aortic root diameter; Lad, Left atrial diameter; LVEDD, Left ventricular end-diastolic dimension; LVESD, Left ventricular end-systolic dimension; RVOT, Right ventricular outflow tract dimension; FS, Fractional shortening; LVEF, Left ventricle ejection fraction*.

At 3 months after PDA closure, the LAd, LVESD, and LVEDD were no different in comparison to the pre-closure baseline. Also, the LVEF and FS values recovered to pre-closure levels in all patients (Table 2).

### The Correlation of Echocardiography Parameters With LVEF Post-closure

To identify the factors associated with post-closure LV systolic function, stepwise multiple linear regression analysis was conducted. Univariate linear regression analysis showed that pre-closure PDAd (*r* = −0.48, *P* < 0.01), PDAd/AOd (*r* = −0.50, *P* < 0.01), and LAd/AOd (*r* = −0.55, *P* < 0.01) negatively correlated with post-closure LVEF, and pre-closure LVEF (*r* = 0.66, *P* < 0.01) positively correlated with post-closure LVEF (Table 3).

**Table 3:** Comparison of pre-closure echocardiography parameters in patients with or without post-closure LV systolic dysfunction.

	**No LV dysfunction (*n* = 164)**	**LV dysfunction (*n* = 27)**
PDAd (mm)	2.87 ± 0.92	4.21 ± 1.37^*^
AOd (mm)	14.24 ± 3.23	13.94 ± 4.73
Lad (mm)	19.89 ± 3.86	19.69 ± 4.73
LVESD (mm)	21.09 ± 3.83	21.53 ± 5.84
LVEDD (mm)	35.60 ± 6.02	35.20 ± 9.35
RVOT (mm)	17.99 ± 4.40	18.53 ± 3.50
FS (%)	38.70 ± 4.20	39.55 ± 4.89
LVEF (%)	70.53 ± 4.73	67.30 ± 6.12^*^
PDAd/AOd	0.21 ± 0.08	0.33 ± 0.09^*^
LAd/AOd	1.41 ± 0.26	1.54 ± 0.24^*^

* *P <0.05 vs. No LV dysfunction*.

*PDAd, Patent ductus arteriosus diameter; AOd, Aortic root diameter; Lad, Left atrial diameter; LVEDD, Left ventricular end-diastolic dimension; LVESD, Left ventricular end-systolic dimension; RVOT, Right ventricular outflow tract dimension; FS, Fractional shortening; LVEF, Left ventricle ejection fraction; PDAd/AOd, the ratio of PDA diameter to aortic root diameter; LAd/AOd, the ratio of left atrial diameter to aortic root diameter*.

Among these parameters, pre-closure LVEF (β = 0.443, *P* < 0.01), PDAd/AOd (β = −0.216, *P* < 0.01), and LAd/AOd (β = −0.211, *P* < 0.01) were statistically significant factors on multivariate stepwise linear regression analyses for the deterioration of post-closure LV (Table 4).

**Table 4:** Multiple linear regression analysis for post-closure LV dysfunction.

**Pre-closure variables**	**Univariate analysis *P***	**Multivariate analysis *P***
AOd (mm)	0.570	
LAd (mm)	0.360	
PDAd (mm)	0.003	
LVESD (mm)	0.105	
LVEDD (mm)	0.192	
ROVT (mm)	0.293	
LVEF (%)	0.003	0.001
PDAd/AOd	0.001	0.001
LAd/AOd	0.001	0.001

*AOd, Aortic root diameter; Lad, Left atrial diameter; LVEDD, left ventricular end-diastolic dimension; LVESD, left ventricular end systolic dimension; ROVT, Right ventricular outflow tract dimension; FS, fractional shortening; LVEF, Left ventricle ejection fraction; PDAd/AOd, the ratio of PDA size to aortic root diameter; LAd/AOd, the ratio of left atrial diameter to aortic root diameter*.

### The Cutoff Value of Echocardiography Parameters Associated With Left Ventricular Dysfunction

**Figure 1:**
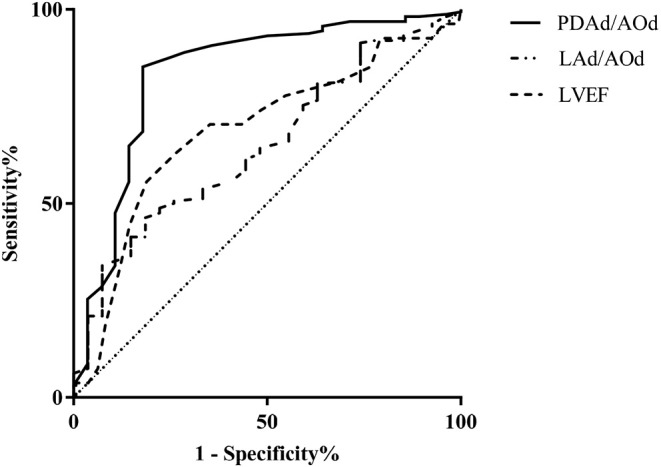
Receiver operating characteristic curve of pre-closure LVEF, PDAd/AOd, and LAd/AOd echocardiography parameters for predicting LV systolic dysfunction.

By ROC analysis, the area under the curve (AUC) for pre-closure PDAd/AOd was 0.833, and pre-closure PDAd/AOd ≥ 0.28 showed a sensitivity of 81.5% and specificity of 98.5%. Moreover, pre-closure LAd/PDAd ≥ 1.54 showed a sensitivity of 81.5% and specificity of 68.5%. Its AUC was 0.692, whereas pre-closure LVEF ≤ 66.5% showed a sensitivity of 81.3% and specificity of 57.5% in predicting the immediate post-closure LV systolic dysfunction, and its AUC was 0.693 (Figure 1).

## Discussion

PDA is a relatively common congenital heart defect. The incidence of PDA is ~1 per 2,000 live births in full-term newborns, and accounts for 5–10% of all congenital heart diseases (17). Now, the prevalence rate of PDA is 0.78 per 1,000 in China according to a recent report (18). Since the first transcatheter PDA closure was conducted by Porstmann et al. in 1967 (19), there have been many significant developments in PDA closure. Now, transcatheter PDA occlusion has become the priority choice to treat most PDA in both children and adults (5). The current study demonstrated an early deterioration of LV function following successful transcatheter ductal closure, which recovered completely within 3 months post-closure. Moreover, pre-closure PDAd/AOd, LAd/AOd, and LVEF were the predictors of post-closure LV dysfunction.

As is known, PDA frequently results in left ventricular volume overload, which is required to increases left ventricular output by Frank-Starling response, and it can therefore overcome the left-to-right shunt and maintain systemic circulation (20, 21). Because the left ventricular remodeling is caused by a significant left-to-right shunt through PDA, it is conceivable that left ventricular reverse remodeling occurs after ductal closure. In the current study, the LAd, LVESD, LVEDD, FS, and LVEF significantly decreased in the 24 h after PDA closure. Consistently, Gupta et al. also found there was a significant reduction in LVEDD and LVESD in immediate post-closure as compared to pre-closure baseline (22); these results confirmed that early PDA closure in childhood may benefit the remodeling of LV.

In 2005, Eerola et al. demonstrated that changes in LV systolic function were caused by PDA closure in children (9). Similarly, Galal et al. found that the closure of relatively large PDA led to a significant immediate deterioration of LV systolic performance in children (23). Consistent with these previous reports, the current study demonstrated that LVEF reduced immediately 24 h after PDA closure, which indicated impaired LV systolic function. The possible explanation for this is that when a hemodynamically significant PDA is closed it abolishes the left-to-right shunt, thereby reducing the preload of the LV. However, it also increases the afterload by eliminating the low-resistance pulmonary circulation from LV outflow circulation. Due to a phenomenon called “afterload mismatch,” this simultaneous reduction in the LV preload and increase in the afterload may lead to LV systolic dysfunction (24).

Furthermore, all of the LV dysfunction recovered to baseline in the follow-up periods in the current study, which is consistent with previous children studies (9, 23). However, about 11% of transcatheter PDA adult patients showed the persistent long-term deterioration of LV systolic function, according to Jeong's study (6). This discrepancy is probably due to the longer duration of volume overload and consequently more extensive and irreversible changes in LV in adults compared to children.

In a study conducted by Agha et al., PDAd was proved to be the predictor of post-closure LV systolic function (25). However, the current study found that PDAd/AOd is a predictor of post-closure LV systolic dysfunction, rather than the absolute diameter of PDA. This inconsistency may be due to age, which can affect the PDA size; “PDA size” is also a relative term with no standardization attached to it, which may limit its practical value somewhat. “PDAd/AOd,” therefore, is a better indicator in predicting post-closure LV systolic function, and these results suggest that the larger the PDAd/AOd value the more likely post-closure LV dysfunction would be. Interestingly, the LAd/AOd ratio also predicted the post-closure LV dysfunction in our study. As is known, a significant hemodynamic shunt via a PDA leads to the enlargement of the left heart and a decreased LV ejection fraction (26). LAd/AOd may thus reflect the severity of ductal shunting in these PDA patients; Iyer et al. also reported that a LAd/AOd of >1.4:1 was associated with a hemodynamically significant ductal flow in a preterm infant (27). Our results suggest that PDA closure should be conducted before LAd/AOd reaches more than 1.54 to achieve a normal LVEF after PDA closure.

The current study found that the incidence of LV systolic dysfunction was 14.1% in this cohort (*n* = 27/191). This is comparable with the studies conducted by Jeong et al. (6) (11.1%), Kim et al. (28) (18.6%) on the Korean population, and Eerola et al. (9) (15.2%) on the Finland cohort. However, it is much lower than a study conducted by Kiran et al., which reported the incidence of LV dysfunction is 22.8% in Indian PDA patients (29). These studies suggest the influencing factors of LV systolic dysfunction may also involve ethnicity and social factors in addition to the heart hemodynamics parameters.

The present study had some limitations. Firstly, this study was retrospective and in a single center despite having a relatively large sample size. Secondly, the study only involved the assessment of the systolic function of the LV by two-dimension echocardiography method.

In conclusion, transcatheter closure of PDA is associated with reversible LV systolic dysfunction in children patients. Pre-closure PDAd/AOd ≥ 0.28, LAd/AOd ≥ 1.54, and LVEF ≤ 66.5%, measured by echocardiography, were the cutoff values used to predict post-PDA closure LV systolic dysfunction. The results of the present study can provide a useful and convenient strategy to predict who (of the patients undergoing PDA device closure) is likely to have LV dysfunction after PDA closure in clinical practice.

## Data Availability Statement

The datasets generated for this study are available on request to the corresponding author.

## Ethics Statement

The studies involving human participants were reviewed and approved by the ethics committee of the Children's Hospital of Soochow University. Written informed consent to participate in this study was provided by the participants' legal guardian/next of kin.

## Author's Note

The authors declare that neither this manuscript nor any similar paper, in whole or in part, have been or will be submitted to or published in any other scientific journal.

## Author Contributions

MH, WQ, and LS conceived and designed the study and analyzed data and wrote the manuscript. MH, WQ, BW, WZ, JZ, YD, QX, JH, JS, LC, HL, and LS performed this study. BW, WZ, JZ, YD, QX, JH, JS, LC, and HL reviewed and edited the manuscript. All authors read and approved the manuscript.

### Conflict of Interest

The authors declare that the research was conducted in the absence of any commercial or financial relationships that could be construed as a potential conflict of interest.
