# Supplementary material for: Echocardiographic Prediction of Left Ventricular Dysfunction After Transcatheter Patent Ductus Arteriosus Closure in Children
Source: Front Pediatr. 2019 Oct 15;7:409. doi: 10.3389/fped.2019.00409 (PMC6812610; doi:10.3389/fped.2019.00409)
Supplement: Supplementary file 1 [file Table_1.DOCX]

**Supplemental Table 1．Comparison of heart rate and blood pressure at pre-closure and post-PDA closure (n=191)**

|  | Pre-closure | Post-closure  24 hours |
| --- | --- | --- |
| Heart rate (bpm) | 112.24±25.45 | 113.55±19.35 |
| SBP (mmHg) | 86.48±17.28 | 85.54±14.24 |
| DBP (mmHg) | 58.56±11.25 | 60.13±13.12 |

SBP: systolic blood pressure, DBP: diastolic blood pressure.
